# Insights into Human Cystic Echinococcosis in the Kurdistan Region, Iraq: Characteristics and Molecular Identification of Cysts

**DOI:** 10.3390/pathogens11040408

**Published:** 2022-03-27

**Authors:** Araz Ramadhan Issa, Sardar Hassan Arif, Ayad Ahmad Mohammed, Federica Santolamazza, Azzurra Santoro, Wijdan Mohammed Salih Mero, Adriano Casulli

**Affiliations:** 1 Department of Biology, Faculty of Science, University of Zakho, Zakho 42002, Kurdistan Region, Iraq; araz.issa@uoz.edu.krd (A.R.I.); wijdan.mero@uoz.edu.krd (W.M.S.M.); 2 Department of Surgery, College of Medicine, University of Duhok, Duhok 42001, Kurdistan Region, Iraq; sardar.arif@uod.ac (S.H.A.); ayad.mohammed@uod.ac (A.A.M.); 3 WHO Collaborating Centre for the Epidemiology, Detection and Control of Cystic and Alveolar Echinococcosis, Department of Infectious Diseases, Istituto Superiore Di Sanità, 00161 Rome, Italy; federica.santolamazza@iss.it (F.S.); azzurra.santoro@iss.it (A.S.); 4 European Union Reference Laboratory for Parasites, Department of Infectious Diseases, Istituto Superiore Di Sanità, 00161 Rome, Italy; 5 College of Science, Nawroz University, Duhok 42001, Kurdistan Region, Iraq

**Keywords:** *Echinococcus granulosus*, G1/G3 genotypes, human cystic echinococcosis, clinical and molecular epidemiology, Kurdistan region, Iraq

## Abstract

Cystic echinococcosis (CE) is a neglected worldwide distributed parasitic disease caused by the *Echinococcus* *granulosus* *sensu lato* (*s.l.*) species complex. For a better understanding of the pathways of transmission of this parasite, clinical and molecular epidemiological studies are particularly needed from endemic areas where data are scant, such as in the Middle East. The study aimed to identify the characteristics, location, cyst stage and species/genotypes of *E.* *granulosus s.l.* complex in humans from the Kurdistan region, Iraq. To this aim, from June 2019 to February 2021, 64 echinococcal cysts were surgically removed from 62 patients in Azadi and Vajeen reference Hospitals at Duhok city, Duhok governorate (Kurdistan region, Iraq). The results confirmed the liver as the most common anatomical site of CE with 72.58% of the cases, followed by the lungs in 19.35%, while 66.13% of CE cases were females. The highest rate of infections occurred in the age class 21–30 (27.42%). High rates of CE were reported among patients living in rural areas and housewives, which were 54.84% and 43.55% of the CE patients, respectively. The fertility of echinococcal cysts was 82.81%, and the viability of fertile protoscoleces was 70.53%. Cysts were staged with ultrasound according to the WHO-IWGE classification as 32.8% CE1, 32.8% CE2, 7.8% CE3a, 9.4% CE3b, 15.6% CE4 and 1.6% CE5. Molecular analyses using mitochondrial NAD5 gene showed that all analyzed samples (*n* = 59) belonged to the genotypes G1 or G3 of *E. granulosus* *sensu stricto* (*s.s.*), thus, confirming sheep–dog–human transmission in the Kurdistan region, Iraq. No statistically significant correlation was found between the genotypes G1–G3 of *E. granulosus s.s.* and variables, such as the fertility, location and cyst stage classification. Based on the present findings, it is necessary to implement monitoring and control programs in sheep and dog populations to decrease the odds of human infections. Public health education campaigns are required to be implemented at the community level to reduce the risk of acquiring CE in humans in the Kurdistan region, Iraq.

## 1. Introduction

Cystic Echinococcosis (CE) is a worldwide zoonotic parasitic disease caused by the larval stage of *Echinococcus granulosus sensu lato* (*s.l.*) infecting humans and animals [1,2]. The adult parasitic stage (tapeworm) resides in the small intestine of dogs and other wild canid definitive hosts, which contaminate the environment with infective eggs contained in their feces. The asexual larva (metacestode) is found in a wide variety of ungulate intermediate hosts, including humans (due to the ingestion of infective eggs) that act as dead-end hosts [3]. CE may develop in any organ or tissue of humans and intermediate hosts, even if most of the infections can be found in the liver and secondarily in the lungs [4].

The molecular identification of species and genotypes causing human CE is important for confirmatory diagnostic purposes, for understanding the routes of parasite transmission and finally to implement targeted control programs. CE is caused by *E. granulosus s.l.*, which is a complex of cryptic species. To date, the genotypes G1, G3, G4, G5, G6/7 cluster, G8 and G10 have been recognized within *E. granulosus s.l.* [5,6,7]. 

These genotypes have been clustered into four different species: *E. granulosus sensu stricto* (*s.s.*) (G1 and G3; previously referred to as the “sheep strain” and “buffalo strain”, respectively), *Echinococcus equinus* (G4; previously referred to as the “horse strain”), *Echinococcus ortleppi* (G5; previously referred to as the “ cattle strain”), *Echinococcus canadensis* G6/7 cluster (previously referred to as the “camel strain” and “pig strain”, respectively), *Echinococcus canadensis* G8 and G10 (also known as the “cervid strains”) and *Echinococcus felidis* (also known as the “lion strain”) [8]. 

Within *E. granulosus s.l.* species complex, *E. granulosus s.s.* (G1 and G3) is considered the most relevant species of public health interest, since it causes vast majority (88.5%) of molecularly confirmed human cases [9]. In fact, very few molecularly confirmed human cases were identified as *E. equinus* (G4), *E. ortleppi* (G5) and *E. canadensis* (G8 and G10) [9,10,11]. *Echinococcus canadensis* (G6/7) transmission to humans plays a more important role than previously considered as it caused around 11% of all documented cases that were genetically confirmed [9].

Cystic echinococcosis is a public health concern of global relevance, including Iraq, where few epidemiological data have been published. Previous studies indicated this area as endemic for *E. granulosus*, such as the cities of Erbil [12], Baghdad [13], Duhok [14] and Mosul [15]. CE surgical incidences of 2.8/100,000, 2/100,000 and 6.3/100,000 inhabitants were reported from Erbil province (Kurdistan region) [12,16,17] with 4.5/100,000 from Basrah province (Iraq) [18] and 5.6/100,000 from Slemani province (Kurdistan region) [19].

Regarding the molecular identification of *Echinococcus* spp., only *E. granulosus s.s.* (G1 and G3 genotypes) and *E. canadensis* (G6 genotype) has been identified thus far in Iraq. In fact, in Duhok province (Kurdistan region), G1 and G3 were reported in humans [20]. In Erbil province (Kurdistan region), G1 and some of its microvariants were reported in sheep and cattle [21,22]. In Slemani province (Kurdistan region), G1 and G3 were detected in humans and domestic animals [23,24]. In Kirkuk province (Iraq), G1 was reported in humans, sheep, goats and cattle [25]. In Misan province (Iraq), G1 and G3 has been recorded in humans, buffaloes, cattle, goats, sheep and camels [26]. In Al-Qadisiyah, Al- Najaf and Al-Diwaniyah provinces (Iraq), G1, G3 and G6 has been also detected in humans, sheep, cattle and camels [27].

The primary aim of this study was to determine the frequency, location and causative species of the echinococcal cysts isolated from humans in the Duhok governorate using a molecular method [28], validated according to regulation ISO/IEC 17,025 [29] for the identification of species and genotypes belonging to *E. granulosus s.l.* The secondary aim of this study was to search for correlations between the genotype and anatomical location, stage and size of echinococcal cysts and the presence of specific potential risk factors for humans.

## 2. Results

### 2.1. Characteristics of Echinococcal Cysts and Patients Affected by CE

**Figure 1:**
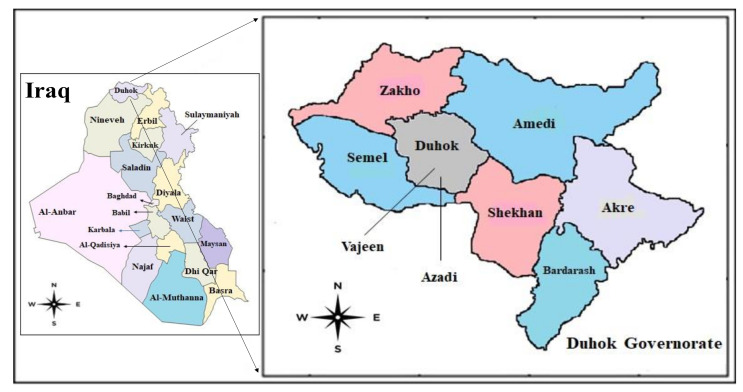
Map of the studied area with Azadi and Vajeen reference Hospitals in Duhok city, Duhok governorate, Kurdistan region, Iraq (from Wikivoiage, 2019).

The present study identified 64 cysts from 62 patients, who underwent surgical operations for confirmed CE from government and private hospitals in Duhok city (Figure 1).

Table 1 shows that 82.81% (53/64) of the isolated parasitic cysts from different organs were fertile, and 17.19% (11/64) were sterile, while calcified cysts were not recorded. The mean viability of fertile cysts was 70.53%. Echinococcal cysts were staged at ultrasound according to the WHO-Informal Working Group on Echinococcosis (WHO-IWGE) classification as follows: 32.8% CE1 (*n* = 21), 32.8% CE2 (*n* = 21), 7.8% CE3a (*n* = 5), 9.4% CE3b (*n* = 6), 15.6% CE4 (*n* = 10) and 1.6% CE5 (*n* = 1).

The characteristics of the patients enrolled in the study are shown in Table 2. According to sex, the rate of infection in females was higher than that of males (66.13% vs. 33.87%). The highest rate of infection was among the age class of 21–30 years (27.42%), while the lowest rate was recorded among the ages 5–10 years (4.84%). According to the anatomical location, the present study revealed that the liver was the predominant site of infection as 71.88% of the parasitic cysts were isolated from there, followed by the lungs at 21.88%. Other sites of infection were the heart (3.12%), spleen (1.56%) and kidney (1.56%).

Regarding multiple organ involvement, two patients were detected with liver–lungs and lung–heart cysts. With respect to the number of echinococcal cysts per patient in the involved organs, for the liver, it was from 1 to 15 echinococcal cysts, while for lungs, it was from one to five cysts. Regarding the symptoms of patients, abdominal pain was detected for cysts localized in the liver (69.56%; 32/46), while chest pain (28.57%; 4/14) was found for cysts in the lungs (Table 3). 

With regard to the occupation, the highest infection rate was recorded among housewives in 43.55% (27/62), followed by students and farmers (both 22.58%). Regarding the residency, most of the infected patients were from rural or semi-rural areas surrounding Duhok city with 54.84% (34/62) and 45.16% (28/62), respectively. According to the size of cysts, most were of medium size (5–10 cm), then large (>10 cm) and small sizes (<5 cm) at rates of 64.52%, 32.26% and 3.22%, respectively (Table 3).

### 2.2. Molecular Analysis of Echinococcal Cysts

A total of 62 cyst samples from 60 patients (two with double organ localization) were molecularly analyzed for *Echinococcus* species identification. Sixty-two cyst samples were analyzed using a Restriction Fragment Length Polymorphism–Polymerase Chain Reaction (PCR-RFLP) assay [28], which resulted in 59 amplifications, while three samples did not amplify. The first step of the method in conventional PCR amplified a fragment of 444 bp, the cytochrome c oxidase subunit 1 (COXI) mitochondrial gene, while the second step (RFLP) produced two fragments of 235 and 209 bp.

All 59 samples were identified as *E. granulosus s.s.* (genotypes G1 and G3), and thus it was not necessary to proceed with the third step of the method (Multiplex PCR), which allows the identification of the remaining genotypes of *E. granulosus s.l*. complex [28]. Fifty-nine *E. granulosus s.s.* samples were further characterized by the amplification and sequencing of the NADH dehydrogenase subunit 5 (NAD5) mitochondrial gene to differentiate genotypes G1 from G3 [30]. Analysis of the informative nucleotide positions within this gene allowed the identification of 47 samples as G1 and 12 as G3. In case of multiple organ involvement, the genotypes were concordant. The nucleotide substitutions are shown in Table 4.

No statistical correlation (*p* > 0.05) was found in the logistic regression between both genotypes G1 and G3 of *E. granulosus s.s.* and the characteristics of the echinococcal cysts, such as fertility, anatomical location and cyst stage according to WHO-IWGE classification.

## 3. Discussion

Even though CE is a serious parasitic disease of increasing public health concern, current global efforts to control the disease are insufficient, as little advances have been made for improving the diagnostics, drugs and clinical management [31,32]. In Iraq, including the Kurdistan region, several epidemiological factors contribute to the transmission of this parasitic infection, such as unauthorized home slaughtering of animals (especially during religious festivals and national holidays), the low level of health education of most butchers and farmers and the presence of stray dogs in the region.

According to this study, the frequency of human fertile cysts was higher than that reported for sterile cysts. This finding highlights the risk of secondary CE infections during surgical procedures, stressing the use of albendazole as an adjuvant either preoperatively or postoperatively. These results are consistent with Salem [33] in Mauritania, Al-Bosely [34] in Zakho (Kurdistan region, Iraq), Piccoli [35] in Romania and Khalf [13] in Baghdad (Iraq) as they reported fertility rates of 76%, 90%, 92% and 47%, respectively. The viability of the protoscoleces reported in this study was 70.53%; similarly, in Zakho city a high viability was reported by Al-Bosely with 79.13% [34]. The fertility rate of hydatid cysts coupled with molecular typing studies are important factors in the epidemiological studies as these could provide valuable information on the pathways of transmission.

Regarding gender, the rate of CE was higher in females compared with in males, this agrees with other studies performed in the Kurdistan region and other parts of Iraq, such as in Theqar [36], Erbil [16], Baghdad [13] and Duhok [14]. All of these studies, when gender data was available, reported higher infection rates in females (vs. males), which were 58.3% (vs. 41.7%), 63.08% (vs. 37.58%), 60% (vs. 40%) and 64.6% (vs. 35.4%), respectively. Similarly, some neighboring countries, such as Turkey [37], Jordan [38] and Iran [39] also reported higher rates of CE among females (8.1%, 67.4% and 57.95%, respectively). 

As previously discussed, the higher prevalence of CE in females in this study might be related to many factors, such as occupation and cultural habits; in addition, females are more in close contact with infection sources, such as soil or vegetables contaminated with viable eggs of *E. granulosus* from dog feces [40]. Nevertheless, being female cannot be excluded as a confounding factor of living in rural contaminated endemic areas, since large cohort studies on CE did not find any statistically significant difference between the male and female prevalence [41].

Regarding the age of operated CE patients, the highest rate of infection was found among the age class of 21–30 years, while the lowest rate was recorded among the age class of 5–10 years. This finding agrees with a previous study in the Kurdistan region of Iraq, Al Saeed and Almufty [14] in Duhok, where high infection rates were reported among the age class 21–30 years (22.9%). 

On the other hand, the current results disagree with some other studies performed in Iraq and worldwide, as they reported that the highest rate of infection occurred among ages of 30–39 years in Iran, 41–50 years in Erbil, 10–19 years in Baghdad and 30–39 years in Turkey and Italy [13,16,37,42,43]. As previously suggested, CE can be acquired at any age [44]. In fact, the higher rate of CE in young ages agree with Beard [44], who found, in a study conducted in Australia and New Zealand, that the incidence of the disease was halved without altering the age distribution, indicating that adults are relatively susceptible.

In the present study, the infection rate of CE in patients living in rural areas was higher than that reported from urban areas. Similarly, Abdi [45] stated that rural inhabitants were more prone to be infected with hydatid cysts than those living in urban communities. In fact, the life cycle of this parasite is maintained in rural areas, where a large number of stray dogs contaminate the environment with *Echinococcus* eggs, thus, increasing the odds of CE infection in humans [42,43].

The present study showed that the liver and lungs are the predominant infection sites for *E. granulosus*. Similar findings were reported by many studies in the Kurdistan region, other areas of Iraq and worldwide, such as in Molan in Theqar (Iraq) [36], Ranjbar-bahadori in Iran [42], Saida and Nouraddin in Erbil (Kurdistan region, Iraq) [12], Khalf in Baghdad (Iraq) [13], Al Saeed and Almufty in Duhok (Kurdistan region, Iraq) [14] and Khan [46] in Pakistan. 

In fact, the liver acts as the first filter for larval infection, and the lungs act as the second filter. Regarding the residency of enrolled patients, a higher proportion of them were from rural settings. Similarly, many researchers reported the highest rate of CE in rural areas, such as Saida and Nouraddin in Erbil (Kurdistan region, Iraq) [12] and Khan in Pakistan [46]. In this aspect, the present results contradict other studies in Iran [39,42] as they reported higher infection rates in urban populations, with 61% and 89.77%, respectively. 

The identification of *E. granulosus s.s.* in the Kurdistan region is in line with other studies that identified this species as the most prevalent (88.5%) in humans among all molecularly confirmed *E. granulosus s.l.* species worldwide [9]. The subset of cysts analyzed for genotype G1 or G3 distinction revealed the presence of both the genotypes and the predominance of G1 genotype, with no specific correlation with age, gender or occupation. 

The results of this study are similar to previous studies from the Kurdistan region and other areas of Iraq, showing that the G1 genotype is predominant in humans from Sulaimani province [47], Dohuk province [20], Kirkuk province [25], Misan province [26] and Al- Najaf and Al-Diwaniyah provinces [27]. Furthermore, these genotypes were reported in countries neighboring Iraq, such as Turkey [48,49], Iran [50,51], Jordan [52] and Saudi Arabia [53,54,55]. These results are also in line with a recent systematic review that highlighted G1 as the main genotype present in Europe [56].

Whether genotypes G1 and G3 may be of different grades of infectivity and pathogenicity to humans and animal species is currently unclear and requires further systematic epidemiological studies. Even though the distinction between G1 from G3 represents a useful tool for source attribution in the field of molecular epidemiology, this study did not find any evidence for potential correlations between cyst biological features and these genotypes.

## 4. Materials and Methods

### 4.1. Collection of Samples

**Figure 2:**
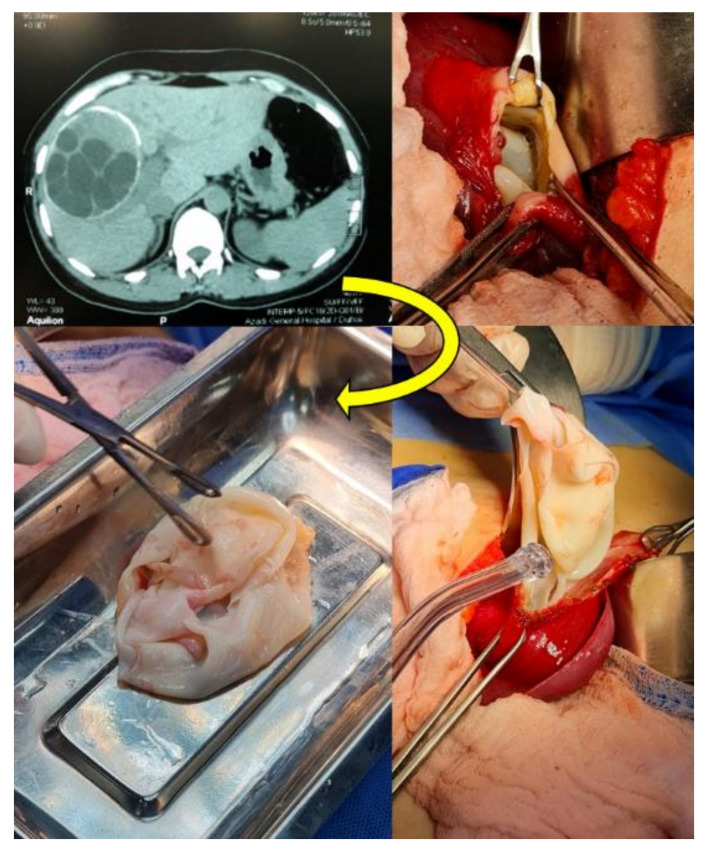
Computed tomography (CT) and the open surgery procedure for the excision of *Echinococcus granulosus sensu lato* cysts (CE3b stage) from a human liver in Duhok city, Duhok governatorate, Kurdistan region, Iraq.

From June 2019 to February 2021, a total of 64 parasitic cysts were isolated and collected from 62 patients admitted in the Azadi and Vajeen reference hospitals in Duhok city (Kurdistan region, Iraq) after removal by an open surgery procedure and pathological confirmation (Figure 1 and Figure 2). Of these 64 echinococcal cysts, 62 were molecularly analyzed. Patient informed consent and ethical committee approval from the Duhok Directorate General of Health (ethical clearance n 1207-2021-7-24) were obtained for the use of data and samples in this study.

Suspected CE cases were clinically examined by physicians and confirmed by one or more imaging diagnostic techniques, such as ultrasound, CT scans and X ray imaging in addition to serology. After surgery, the cysts were confirmed by histopathology [16,17,19].

It should be also emphasized that, among all imaging techniques, ultrasound (US) has a key role in the diagnosis, clinical management and follow-up of patients affected by CE since it is portable and can be used in the field and rural settings, harmless and does not require patient preparation and can be repeated as often as required. Due to US classification, the concept of stage-specific treatments was introduced providing new insight in the natural history of this parasitic disease. In this context, human echinococcal cysts from this study were staged at ultrasound according to the WHO-IWGE [57]. 

Uncomplicated cyst stages CE1, CE2, CE3a and CE3b less than 5 cm were medically treated with Albendazole (10–15 mg/Kg) for 3–6 months with follow-up by US. Cyst stages CE1, CE2, CE3a and CE3b more than 5 cm received surgical intervention with one-month preoperative and 2 months postoperative albendazole. Inactive cyst stages CE4 and CE5, if uncomplicated, received follow-up (the watch and wait approach) after excluding solid organ tumors unless there were complications.

Each isolated parasitic cyst sample was stored in a sterile tube and delivered, within half an hour after surgical excision, to the Advanced Microbiology Laboratory, Biology Department, Faculty of Science (University of Zakho, Zakho city, Duhok governatorate, Kurdistan region, Iraq). Each cyst was divided into two parts: one containing the germinal layer with or without protoscoleces was stored in 70% ethanol at −20 °C for molecular analyses, and the remaining part was examined by microscope to detect the presence of protoscoleces and to determine the rate of viability.

### 4.2. Examination of Fertility and Viability of Echinococcal Cysts

**Figure 3:**
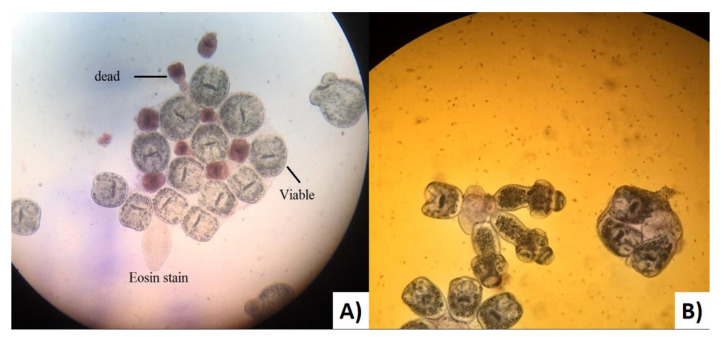
(**A**) Viable and dead protoscoleces of *Echinococcus granulosus* *sensu lato* from the Kurdistan region (Iraq) stained with aqueous Eosin stain. (**B**) Evaginated protoscoleces of *Echinococcus granulosus sensu lato* without stain.

The estimation of the viability rate of fertile echinococcal cysts was performed at the Advanced Microbiology Laboratory, Biology Department, Faculty of Science (University of Zakho). Each cyst fluid was centrifuged for 5 min at 3000× *g* rpm. Subsequently, one drop of the precipitate was taken using a sterile pipette and placed onto a clean glass slide together with a drop of 0.1% aqueous eosin solution (*v*/*v*), mixed and covered with a cover slip and examined under 40× magnification. Living protoscoleces did not take up the stain, unlike the dead ones as shown in Figure 3 [58].

The viability was determined by the following formula:

$$\mathrm{Viability}\;\mathrm{of}\;\mathrm{protoscoleces}\;=\frac{\mathrm{No}\;\mathrm{of}\;\mathrm{viable}\;\mathrm{protoscoleces}\;}{\mathrm{Total}\;\mathrm{No}.\;\mathrm{of}\;\mathrm{protoscoleces}}\times 100$$

### 4.3. Molecular Typing of Echinococcus Granulosus s.l.

Sixty-two out of 64 cysts isolated from the liver, lungs and other organs from humans were delivered to the Department of Infectious Diseases (Istituto Superiore di Sanità, Rome, Italy) where DNA extraction and the molecular identification of cysts were carried out. Genomic DNA from protoscoleces or the germinal layer was extracted using the DNeasy Blood & Tissue kit (Qiagen, Valencia, CA, USA), according to the manufacturer’s instructions. A negative control (nuclease-free water) was included in this working session to verify the absence of contamination during the DNA extraction. DNA was stored at −20 °C until use.

The molecular identification of genotypes/species belonging to *E. granulosus s.l.* was performed according to the recently validated protocol (ISO/IEC 17025) described by Santolamazza and colleagues [28,29]. In brief, the method first identifies the common *E. granulosus s.s*. based on a PCR-RFLP assay and can further identify the remaining genotypes/species based on a multiplex PCR assay (*E. equinus*, *E. ortleppi*, *E. canadensis* G6/7 and *E. canadensis* G8/G10). 

From the echinococcal cysts identified as *E. granulosus s.s.,* discrimination between genotypes G1 and G3 was further achieved by sequencing the NAD5 gene. Amplification of the NAD5 gene (amplicon size 759 bp) and genotype discrimination based on Single-Nucleotide Polymorphism (SNP) analysis were performed according to the protocol described by Kinkar and colleagues [30]. 

The correlations between *E. granulosus s.s.* genotypes G1 and G3 and cyst-related variables (anatomical location, fertility and cyst stage according to the WHO-IWGE classification) were investigated using logistic regression in univariate and multivariate analysis with the genotype considered as a dichotomous outcome. Statistical analysis was performed using Statistical Package for the Social Sciences (SPSS; version 26, 2019) [59]; *p* values < 0.05 were considered as significant.

## 5. Conclusions

In conclusion, this study confirmed that CE is endemic with higher rates in rural areas and among females, and it is caused by *E. granulosus s.s*. in the Duhok, Kurdistan region of Iraq. Based on the present findings, it is necessary to implement monitoring and control programs in sheep and dog populations to decrease the odds of human infections. This requires public health education campaigns implemented at the community level. Awareness hygiene campaigns on how to properly wash vegetables, fruits and hands and how to dispose of infected sheep offal should be considered as the main control measures to reduce the risk for acquiring CE in humans in the Kurdistan region of Iraq.

## Figures and Tables

**Table 1:** The number and fertility rate of human cysts isolated from surgical procedures according to anatomical locations in the Duhok governorate, Kurdistan region (Iraq).

Cyst Location	No. of Examined Cysts	Type of Cysts	
		Fertile (%)	Sterile (%)
Liver	46	38 (82.61)	8 (17.39)
Lung	14	12 (85.71)	2 (14.29)
Heart	2	1 (50)	1 (50)
Kidney	1	1 (100)	--
Spleen	1	1 (100)	--
**Total**	**64**	**53 (82.81)**	**11 (17.19)**
Viability rate	10 (70.53)		

**Table 2:** Characteristics of the 62 patients with cystic echinococcosis enrolled in the study from the Duhok governorate, Kurdistan region, Iraq.

Parameters	Factors	Number	%
Sex	Female	41	66.13
	Male	21	33.87
Age group in years	5–10	3	4.84
	11–20	11	17.74
	21–30	17	27.42
	31–40	13	20.97
	41–50	7	11.29
	51–60	4	6.45
	61–80	7	11.29
Cyst location	Liver	46	71.88
	Lung	14	21.88
	Heart	2	3.13
	Kidney	1	1.56
	Spleen	1	1.56
Occupation	Housewives	27	43.55
	Student	14	22.58
	Farmer	14	22.58
	Soldier	4	6.45
	No job	2	3.23
	Retired	1	1.61
Residency	Urban	28	45.16
	Rural	34	54.84
Cyst size	Large (>10 cm)	20	32.26
	Medium (5–10 cm)	40	64.51
	Small (<5 cm)	2	3.23

**Table 3:** Summary of the symptoms experienced by 62 patients that lead them to seek medical advice and discover that they were infected with cystic echinococcosis in the Kurdistan region, Iraq.

Anatomical Location	Number of Patients	Symptoms (If Present)
Liver	32	Abdominal pains
	9	Incidental finding
	1	Jaundice
	2	Shortness of breath
	1	Acute Abdomen, hypotension
	1	Fever
Lung	4	Chest pain
	7	Shortness of breath
	2	Incidental finding
	1	Hemoptysis
Heart	1	Shortness of breath
	1	Incidental finding
Kidney	1	Incidental finding
Spleen	1	Abdominal pain

**Table 4:** The number and percentages of G1 and G3 *E. granulosus sensu stricto* samples (*n* = 59) from the Kurdistan region, Iraq, identified in the current study and their anatomical location. Nucleotide substitutions of the NAD5 mitochondrial gene, based on the Reference G1 sequence AB786664 are shown.

Genotype	Number (%)	NAD5 Nucleotide Substitutions						Location			
		758	781	1035	1123	1371	1380	Liver	Lung	Heart	Kidney
								*n* (%)	*n* (%)	*n* (%)	*n* (%)
G1	47 (79.7)	G	A	C	G	A	G	32 (54.2)	12 (20.3)	2 (3.4)	1 (1.7)
G3	12 (20.3)	C	G	T	A	G	A	10 (16.9)	2 (3.4)	0 (0.0)	0 (0.0)
**Total**	59 (100)							42 (71.2)	14 (23.7)	2 (3.4)	1 (1.7)

## Data Availability

Materials described in the manuscript, including all relevant raw data, are freely available.

## References

[1] Deplazes P., Rinaldi L., Rojas C.A., Torgerson P.R., Harandi M.F., Romig T., Magambo J. (2017). Global distribution of alveolar and cystic echinococcosis. Advances in Parasitology.

[2] Romig T., Deplazes P., Jenkins D., Giraudoux P., Massolo A., Craig P.S., De La Rue M. (2017). Ecology and life cycle patterns of *Echinococcus* species. Advances in Parasitology.

[3] Thompson R.C.A. (2008). The taxonomy, phylogeny and transmission of *Echinococcus*. Exp. Parasitol..

[4] Gottstein B., Reichen J. (2002). Hydatid lung disease (echinococcosis/hydatidosis). Clin. Chest Med..

[5] Nakao M., Yanagida T., Konyaev S., Lavikainen A., Odnokurtsev V.A., Zaikov V.A., Ito A. (2013). Mitochondrial phylogeny of the genus *Echinococcus* (Cestoda: Taeniidae) with emphasis on relationships among *Echinococcus canadensis* genotypes. Parasitology.

[6] Romig T., Ebi D., Wassermann M. (2015). Taxonomy and molecular epidemiology of *Echinococcus granulosus sensu lato*. Vet. Parasitol..

[7] Vuitton D.A., McManus D.P., Rogan M.T., Romig T., Gottstein B., Naidich A., Tuxun T., Wen H., Menezes Da Silva A. (2020). The World Association for Online Education International consensus on terminology to be used in the field of *Echinococcoses*. Parasite.

[8] Nakao M., Yanagida T., Okamoto M. (2010). State-of-the-art *Echinococcus* and *Taenia*: Phylogenetic taxonomy of human-pathogenic tapeworms and its application to molecular diagnosis. Infect. Genet. Evol..

[9] Alvarez Rojas C.A., Romig T., Lightowlers M.W. (2014). *Echinococcus granulosus sensu lato* genotypes infecting humans—Review of current knowledge. Int. J. Parasitol..

[10] Kim H.J., Yong T.S., Shin M.H., Lee K.J., Park G.M., Suvonkulov U., Kovalenko D., Yu H.S. (2020). Phylogenetic characteristics of *Echinococcus granulosus sensu lato* in Uzbekistan. Korean J. Parasitol..

[11] Macin S., Orsten S., Samadzade R., Colak B., Cebeci H., Fındık D. (2021). Human and animal cystic echinococcosis in Konya, Turkey: Molecular identification and the first report of *E. equinus* from human host in Turkey. Parasitol. Res..

[12] Saida L.A., Nouraddin A.S. (2011). Epidemiological study of cystic echinococcosis in Man and slaughtered Animals in Erbil province, Kurdistan Regional-Iraq. Tikrit J. Pure Sci..

[13] Khalf M.S., AlTaie L.H., AlFaham M.A. (2014). The incidence of hydatid cyst in human in baghdad governorate. IOSR J. Pharm. Biol. Sci..

[14] Al Saeed A.T.M., Almufty K.S.A. (2016). Human hydatidosis in Duhok–Kurdistan Region–North of Iraq. Med. J. Babylon.

[15] Al-Mukhtar A., Qasim I.K. (2017). Serological survey of hydatid disease in asymptomatic peoples in Mosul City, Iraq. Rafidain J. Sci..

[16] Al-Barwari S.E., Saeed I.S., Khalid W., Al-Harmni K.I. (1991). Human hydatidosis in Arbil, N. Iraq. J. Islamic Acad. Sci..

[17] Saeed I., Kapel C., Saida L.A., Willingham L., Nansen P. (2000). Epidemiology of *Echinococcus granulosus* in Arbil Province, Northern Iraq, 1990–1998. J. Helminthol..

[18] Abdulhameed M.F., Habib I., Al-Azizz S.A., Robertson I. (2018). A retrospective study of human cystic echinococcosis in Basrah Province, Iraq. Acta Trop..

[19] Barzanji A.A., Saida L.A. (2019). Echinococcosis in Kurdistan Iraq: Prevalence of cystic hydatidosis in man with a survey of *E. granulosus* eggs in stray dogs in Kalar City, Sulaymania Province, Kurdistan–Iraq. J. Univ. Raparin.

[20] Ahmed B.D., Mero W.M.S., Salih A.M., Xiao N., Casulli A., Abdo J.M. (2013). Molecular characterization of *Echinococcus granulosus* isolated from human hydatid cyst using mitochondrial *Cox1* gene sequencing in Dohuk Province–Kurdistan Region, Iraq. Sci. J. Univ. Zakho.

[21] Hassan Z.I., Meerkhan A.A., Boufana B., Hama A.A., Ahmed B.D., Mero W.M.S., Orsten S., Maria Interisano M., Pozio E., Casulli A. (2017). Two haplotype clusters of *Echinococcus granulosus sensu stricto* in Northern Iraq (Kurdistan Region) support the hypothesis of a parasite cradle in the Middle East. Acta Trop..

[22] Abdulla R.G., Mageed S.N., Obed C.E., Jumaa J.A. (2020). Molecular characterization of fertile hydatid cysts from the liver of the sheep and cows and associated environmental influence factors. Iraqi J. Vet. Sci..

[23] Hama A.A., Hassan Z.I., Mero W.M.S., Interisano M., Boufana B., Casulli A. (2015). A morphologically unusual *Echinococcus granulosus* (G1 Genotype) cyst in a cow from Kurdistan–Iraq. Epidemiology.

[24] Hama A.A., Mero W.M.S., Jubrael J.M.S. Genotyping of *Echinococcus granulosus* (Hydatid Cyst) isolated from domestic animals in Kurdistan–Iraq. Proceedings of the International Conference on Pure and Applied Sciences (ICPAS 2018).

[25] Hassan H.F., Fadhil M.H., Fadhil Z.H. (2016). Molecular characterization of *Echinococcus granulosus* isolated from human and domestic animals in Kirkuk, Iraq. Anim. Res. Int..

[26] Alsaady H.A.M., Al-Quzweeni H.A.N. (2019). Molecular study of *Echinococcus granulosus* in Misan Province, South of Iraq. Indian J. Public Health Res. Dev..

[27] Mahdi Z.M.S., Al-Hamairy A.K., Al-Rubaiey H.M. (2020). Genotyping of *Echinococcus granulosus* isolates from human, sheep and cattles hydatid cysts in some Central Euphrates Provinces, Iraq. Med.-Leg. Update.

[28] Santolamazza F., Santoro A., Possenti A., Cacciò M.S., Casulli A. (2020). A validated method to identify *Echinococcus granulosus sensu lato* at species level. Infect. Genet. Evol..

[29] (2017). 2017 General Requirements for the Competence of Testing and Calibration Laboratories, 3rd ed.

[30] Kinkar L., Laurimäe T., Acosta-Jamett G., Andresiuk V., Balkaya I., Casulli A. (2018). Distinguishing *Echinococcus granulosus sensu stricto* genotypes G1 and G3 with confidence: A practical guide. Infect. Genet. Evol..

[31] McManus D.P., Thompson R.C.A. (2003). Molecular epidemiology of cystic Echinococcosis. J. Parasitol..

[32] Casulli A. (2020). Recognising the substantial burden of neglected pandemics cystic and alveolar echinococcosis. Lancet Glob. Health.

[33] Salem O.A., Schneegans F., Chollet J.Y., Jemli M.H. (2011). Epidemiological studies on *Echinococcosis* and characterization of human and livestock hydatid cysts in Mauritania. Iran. J. Parasitol..

[34] Al-Bosely A.R.I. (2013). Studies on epidemiology and some enzyme activities in laminated and germinal layers of hydatid cysts isolated from different intermediate hosts in Zakho, Duhok Province, Kurdistan Region of Iraq. Master’s Thesis.

[35] Piccoli L., Bazzocchi C., Brunetti E., Mihailescu P., Bandi C., Mastalier B., Cordos I., Beuran M., Popa L.G., Meroni V. (2013). Molecular characterization of *Echinococcus granulosus* in South-Eastern Romania: Evidence of G1–G3 and G6–G10 complexes in humans. Clin. Microbiol. Infect..

[36] Molan A.L. (1993). Epidemiology of hydatidosis and echinococcosis in Theqar Province, Southern Iraq. Jpn. J. Med. Sci. Biol..

[37] Akalin S., Kutlu S.S., Caylak S.D., Onal O., Kaya S., Bozkurt A.Y. (2014). Seroprevalence of human cystic echinococcosis and risk factors in animal breeders in rural communities in Denizli, Turkey. J. Infect. Dev. Ctries.

[38] Himsawi N., Hijjawi N., Al-Radaideh A., Al-Tamimi M. (2019). Seroprevalence of cystic echinococcosis in a high-risk area (Al-Mafraq Governorate) in Jordan, using indirect hemagglutination test. Parasite Epidemiol. Control.

[39] Saghafipour A., Divband M., Farahani L.Z., Parsa H.H., Fard H.G. (2020). Epidemiology, burden, and geographical distribution of cystic echinococcosis in Central Iran. Int. J. One Health.

[40] Possenti A., Manzano-Román R., Sánchez-Ovejero C., Boufana B., La Torre G., Siles-Lucas M., Casulli A. (2016). Potential risk factors associated with human cystic echinococcosis: Systematic review and meta-analysis. PLoS Negl. Trop. Dis..

[41] Tamarozzi F., Akhan O., Cretu C.M., Vutova K., Akinci D., Chipeva R., Ciftci T., Constantin C.M., Fabiani M., Golemanov B. (2018). Prevalence of abdominal cystic echinococcosis in rural Bulgaria, Romania, and Turkey: A cross-sectional, ultrasound-based, population study from the HERACLES project. Lancet Infect. Dis..

[42] Ranjbar-bahadori S., Lotfollahzadeh S., Vaezi G., Eslami A. (2008). Epidemiological study of the human cystic echinococcosis in Iran. Res. J. Parasitol..

[43] Manfredi M.T., Di Cerbo A.R., Zanzani S., Moriggia A., Fattori D., Siboni A., Bonazza V., Filice C., Brunetti E. (2011). Prevalence of *Echinococcosis* in humans, livestock and dogs in Northern Italy. Helminthologia.

[44] Beard T.C. (1978). Evidence that a hydatid cyst is seldom ‘As old as the patient’. Lancet.

[45] Abdi J., Taherikalani M., Asadolahi K., Emaneini M. (2013). Echinococcosis/hydatidosis in Ilam Province, Western Iran. Iran. J. Parasitol..

[46] Khan A., Ahmed H., Simsek S., Liu H., Yin J., Wang Y., Shen Y., Cao J. (2020). Molecular characterization of human *Echinococcus* isolates and the first report of *E. canadensis* (G6/G7) and *E. multilocularis* from the Punjab Province of Pakistan using sequence analysis. BMC Infect. Dis..

[47] Hama A.A., Mero W.M.S., Jubrael J.M.S. Molecular characterization of *E. granulosus*, first report of sheep strain in Kurdistan-Iraq. Proceedings of the 2nd International Conference on Ecological, Environmental and Biological Sciences (EEBS 2012).

[48] Vural G., Baca A.U., Gauci C.G., Bagci O., Gicik Y., Lightowlers M.W. (2008). Variability in the *Echinococcus granulosus* cytochrome C oxidase1 mitochondrial gene sequence from livestock in Turkey and a re-appraisal of the G1–3 genotype cluster. Vet. Parasitol..

[49] Kurt A., Avcioglu H., Guven E., Balkaya I., Oral A., Kirman R., Bia M.M., Akyuz M. (2020). Molecular characterization of *Echinococcus multilocularis* and *Echinococcus granulosus* from cysts and formalin-fixed paraffin-embedded tissue samples of human isolates in northeastern Turkey. Vector Borne Zoonotic Dis..

[50] Parsa F., Haghpanah B., Pestechian N., Salehi M. (2011). Molecular epidemiology of *Echinococcus granulosus* strains in domestic herbivores of Lorestan, Iran. Jundishapur J. Microbiol..

[51] Arbabi M., Pirestani M., Delavari M., Hooshyar H., Abdoli A., Sarvi S. (2017). Molecular and morphological characterizations of *Echinococcus granulosus* from human and animal isolates in Kashan, Isfahan Province, Iran. Iran. J. Parasitol..

[52] Issa H.S., Abdel-Hafez S.K., Hijjawi N.S., Al-Qaoud K.M. (2018). Molecular characterization of *Echinococcus granulosus sensu stricto* cysts of domestic ruminants in Jordan. Jordan J. Biol. Sci..

[53] Metwally D.M., Qassim L.E., Al-Turaiki I.M., Almeer R.S., El-Khadragy M.F. (2018). Gene-based molecular analysis of COX1 in *Echinococcus granulosus* cysts isolated from naturally infected livestock in Riyadh, Saudi Arabia. PLoS ONE.

[54] AL-Mutairi N.M., Taha H.A., Nigm A.H. (2020). Molecular characterization of *Echinococcus granulosus* in livestock of Al-Madinah (Saudi Arabia). J. Helminthol..

[55] Al-Hizab F.A., Mohamed N.S., Wassermann M., Hamouda M.A., Ibrahim A.M., Ghareeb W.R., Abdel-Raheem S.M., Romig T., Omer R.A. (2021). Three species of *Echinococcus granulosus sensu lato* infect camels on the Arabian Peninsula. Parasitol. Res..

[56] Casulli A., Massolo A., Saarma U., Umhang G., Santolamazza F., Santoro A. (2022). Species and genotypes belonging to *Echinococcus granulosus sensu lato* complex causing human cystic echinococcosis in Europe (2000–2021): A systematic review approach. Parasites Vectors.

[57] Brunetti E., Kern P., Vuitton D.A. (2010). Writing Panel for the WHO-IWGE. Expert consensus for the diagnosis and treatment of cystic and alveolar echinococcosis in humans. Acta Trop..

[58] Esfahani B., Youssefi M.R. (2010). Comparison of eosin and trepan blue stain in viability of hydatid cyst protoscolices. J. Glob. Vet..

[59] SPSS (2019). Statistical Package for Social Sciences, Version 26.

